# The “myth” of loss of angiogenesis in systemic sclerosis: a pivotal early pathogenetic process or just a late unavoidable event?

**DOI:** 10.1186/s13075-017-1370-5

**Published:** 2017-07-06

**Authors:** Marco Matucci-Cerinic, Mirko Manetti, Cosimo Bruni, Ines Chora, Silvia Bellando-Randone, Gemma Lepri, Amato De Paulis, Serena Guiducci

**Affiliations:** 1 Department of Experimental and Clinical Medicine, Division of Rheumatology and Scleroderma Unit, Azienda Ospedaliera Universitaria Careggi, University of Florence, Viale Pieraccini 18, 50139 Florence, Italy; 2 Department of Experimental and Clinical Medicine, Section of Anatomy and Histology, University of Florence, 50134 Florence, Italy; 3 Department of Internal Medicine, São João Hospital Center, Al Prof Hernâni Monteiro, 4200-319 Porto, Portugal; 4 Department of Translational Medical Sciences, Centre for Basic and Clinical Immunology Research (CISI), University of Naples Federico II, Corso Umberto I, 40, 80138 Naples, Italy

**Keywords:** Scleroderma, Systemic sclerosis, Angiogenesis

## Abstract

Systemic sclerosis is considered a disease dominated by a “loss of angiogenesis”, although in its early phases evidence indicates a disturbed angiogenic response only. In fact, microvascular changes are primarily due to endothelial cell injury, triggering downstream significant *enlargement of the capillary* in an inflammatory environment, followed by capillary rupture (microhemorrhages). Subsequent pro-angiogenic efforts lead to an *aberrant angiogenesis* and, eventually, to a total loss of vessel repair and regeneration (*loss of angiogenesis*). This clearly suggests that the pathogenetic process has a steady progression: from an early excessive pro-angiogenesis, to an aberrant microvascular regeneration, then ending with a late loss of angiogenesis. Herein, we suggest the loss of angiogenesis should not be considered as an overall “myth” characterizing systemic sclerosis but as a very late event of the vascular pathogenesis. Future research should be oriented essentially on the earlier phases dominated by excessive pro-angiogenesis and microvascular aberration.

## Background

In systemic sclerosis (SSc), the loss of angiogenesis has been considered a pivotal event characterizing the disease from its onset. In reality, several pathways of endothelial cell (EC) dysfunction and defective angiogenesis have been identified (Table 1) [1–10]. Paradoxically, in SSc significant concentrations of intrinsic pro-angiogenic factors have been found in the vasculature or adjacent tissues [1, 2, 11]. This evidence is apparently in contrast with the main hypothesis that SSc is fundamentally and originally characterized by a lack or *loss of angiogenesis*. In fact, the eventual inability to regenerate injured vessels may be due to the failure of some angiogenic steps, such as lumen formation or vessel maturation or stabilization. Clearly, the persistent endothelial injury may not only switch on but also significantly perpetuate this process [2]. Whether dysregulated levels of circulating angiogenic or angiostatic factors (or both) are a cause or a consequence of an ongoing vascular disease is still unknown [2].

**Table 1:** Evidence for endothelial cell dysfunction and defective angiogenic pathways in systemic sclerosis

In vitro studies on peripheral blood mononuclear cells suggest a defective contribution of immune cells to angiogenesis [1]. Greater up-regulation of angiostatic than pro-angiogenic mediators [1, 2]. Microarray studies of microvascular EC gene expression have shown an overexpression of either several pro-angiogenic transcripts or many genes that have a negative effect on angiogenesis [1]
Circulating endothelial progenitor cells, involved in postnatal vasculogenesis, are decreased and functionally impaired [1, 5]. Moreover, these cells show mesenchymal properties that may indicate that they potentially contribute to the accumulation of connective tissue and to vascular malfunction [6]
Bone marrow-derived CD14+ monocytic pro-angiogenic hematopoietic cells (promoting vascular formation and repair and differentiation into mural cells) are significantly increased. They can differentiate into fibroblast-like cells producing extracellular matrix proteins contributing to the fibrotic process [7]
Platelet activation contributes to the pro-angiogenic/angiostatic imbalance by release of bioactive factors and aggregation [8]
A change in the endothelial phenotype of residual microvessels is also present in the skin, favoring anti-angiogenic mechanisms [9]
The endothelial-to-mesenchymal transition process is now clarified and is a novel concept in understanding the significant contribution that ECs may play also in the pathogensis of fibrosis [10]

In early SSc pathogenesis, inflammatory cells have a significant role. In fact, in the edematous tissues, where fibrosis is still absent but inflammation dominates, a significant number of immune cells surround the micro-vessels. The role of these cells is pivotal in triggering the activation of cells present in the tissues, in particular myofibroblasts, through the release of TGFβ and other cytokines and growth factors [12]. However, other questions remain unanswered. The first is if autoantibodies are pathogenic, contributing to endothelial damage and thus an expression of disease activity, or just innocent bystanders. The second is if endothelial circulating progenitors as well as the resident mesenchymal cells and the cells surrounding the vessel (pericytes, telocytes) may participate in the endothelial dysfunction and the loss of angiogenesis and foster the endothelial to mesenchymal transition (endoMT) process [4, 10, 13].

**Fig. 1:**
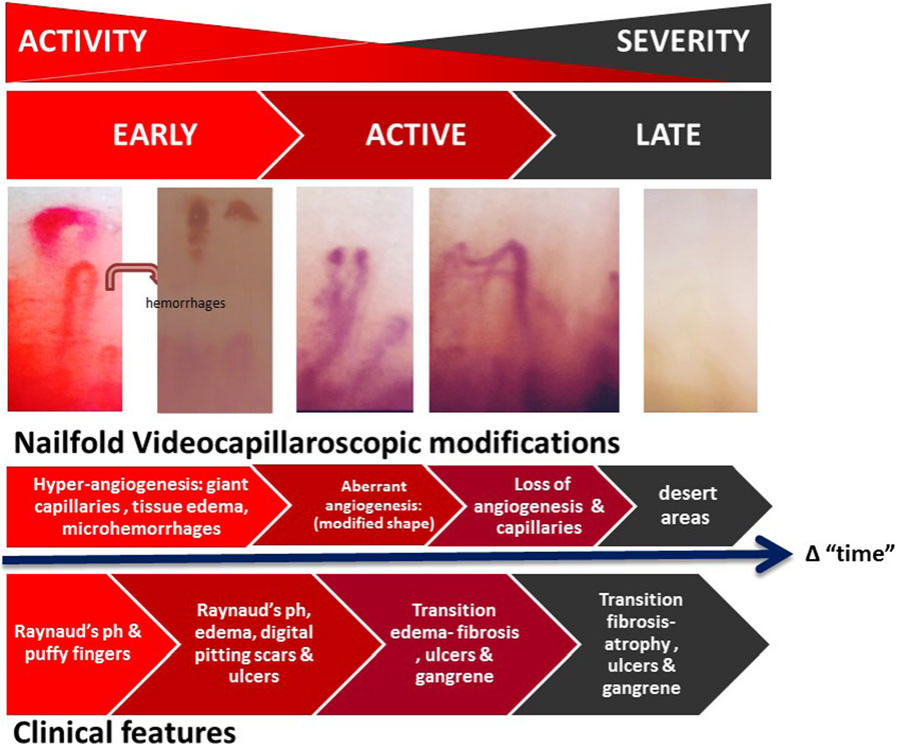
Clinical and microvascular evolution of systemic sclerosis. *Raynaud’s ph* Raynaud’s phenomenon

Angiogenesis is a complex and finely balanced physiological process, consisting of the formation of new vessels from pre-existing ones, mainly triggered by damage or tissue hypoxia [1]. Sprouting angiogenesis encompasses an increase in vasopermeability, leading to plasma and protein extravasation, which works as a temporary scaffold for migrating ECs. Matrix metalloproteinases, secreted by the endothelium, break down the vascular basement membrane and allow the invasion of the surrounding stroma by ECs, directed towards the pro-angiogenic stimulus [1]. This process is paralleled by proliferation and organization of newly formed ECs into three-dimensional tubular structures. Lumen formation and vessel wall stabilization by pericytes are the final phases of sprouting angiogenesis and lead to the creation of a functional network of new capillary vessels [1]. Physiological angiogenesis is finely balanced and regulated by stimulating (pro-angiogenic) and inhibiting (anti-angiogenic) factors [1, 2]. Vascular endothelial growth factor (VEGF) has a key role in controlling several cellular and molecular steps in the angiogenic cascade [1]. Indeed, it stimulates ECs to increase their migration and to initiate the proliferative process until a complete tubular structure is formed [1]. Although the majority of studies in the literature highlight the *loss of angiogenesis* in SSc, in practice this condition is present only in the late phase of SSc evolution (Fig. 1). In fact, in the early stage of the disease a vasculopathy with a pro-inflammatory state is evident and an increased production of pro-angiogenic factors (e.g., VEGF, endothelin-1) has been shown [14, 15], despite the defective response of ECs to these stimuli [16]. Moreover, intrinsic abnormal properties of the cellular components of the blood vessels, the presence of fibroblast-derived anti-angiogenic factors, dysfunctional circulating endothelial progenitor cells, and an abnormal expression of transcription factors, including Fra2 and Fli1, may further contribute to SSc vasculopathy [17, 18].

Vascular abnormalities are indeed manifest very early in SSc [3, 19] and are characterized by a progressive involvement of the vessel wall. The main vascular modifications observed with electron and optical microscopy are EC activation/injury and apoptosis, opening of the EC tight junctions allowing inflammatory cell migration, basal membrane duplication, and intimal thickening with vessel narrowing and obliteration [3].

In the early phase, nailfold videocapillaroscopy shows clusters of giant capillaries and tissue edema surrounded by normal capillaries of different shapes. Micro-hemorrhages, derived from the break of mega-capillaries pushed to their upper limits by an *excessive and uncontrolled angiogenesis*, embedded in an inflammatory environment, are clearly detectable [3]. This *“push” of angiogenesis* and the modification of the capillary shape may depend upon the persistent high circulating levels and tissue over-expression of VEGF [11, 14]. These early vascular changes subsequently lead to vascular tone dysfunction, followed by reduced capillary blood flow, with consequent chronic tissue hypoxia, further exacerbated by extracellular matrix accumulation and fibrosis [3]. The process is then characterized by a profoundly *disturbed and aberrant angiogenesis* (tortuous, ramified, and tree-like capillaries; Fig. 1). This phase is followed by a subsequent microvascular loss—known as *loss of angiogenesis*—which is due to defects in both vascular repair and growth of new vessels through vasculogenesis and angiogenesis [1], characterized by a capillaroscopic progressive reduction in capillary density, with large avascular areas (desertification) (Fig. 1).

This evidence demonstrates the switch from an initial pro- to a final anti-angiogenic environment linked to the preponderant action of angiostatic factors (e.g., VEGF165b, angiopoietin-2), resulting in the loss of new normal vessel formation and capillaries. In the early phase, the hypothesis that increased plasma levels of antiangiogenic VEGF165b isoforms profoundly disturb the pro-angiogenic effect of VEGF, being associated with the severity of capillary architectural derangement and loss, has found significant support [14]. The hypothesis that the initial excessive up-regulation of pro-angiogenic factors might lead to an even greater up-regulation of angiostatic factors is also interesting [2]. The reduction of capillary density consequently leads to an impairment in the supply of oxygen and nutrients and thus to a hypoxic state. In this situation, angiogenesis is usually triggered but in SSc the vascular recovery is profoundly disturbed and then impaired and the *loss of angiogenesis* with avascular areas becomes eventually a prominent event [20–22].

In clinics, SSc is characterized by an evolution which is frequently unpredictable, with abrupt acceleration and periods of quiescence. Therefore, awareness of the condition of the microvasculature in the frame of the disease evolution is crucial and may influence the clinical strategy according to a correct evaluation of the disease phase. In practice, it becomes of paramount importance to establish the real disease phase, which should not be centered on the mere measurement of the years from diagnosis but clearly aimed at understanding the “*real time*” of advancement of the microvascular disease (Fig. 1). In SSc, the evolution of microcirculatory modifications and the time to *loss of angiogenesis* may be very fast in diffuse SSc, while it is significantly slower in limited SSc. A switch of research interest to on the early phase of the disease might change the approach to the clinical setting in SSc. In this perspective, the choice of a vasoactive therapeutic strategy aiming at the modulation, in the “time” frame of each phase of microvascular involvement, of the angiogenic process might be a pivotal event changing the approach to SSc therapy in diffuse or limited SSc. In the future, targeting the early inflammatory pro-angiogenic process [22] leading to capillary aberration might be a relevant step to block the disease evolution to prevent the loss of angiogenesis.

## Conclusions

The fate of SSc is dictated by the phase of evolution of the microvascular modifications observed in the patient [3]. It is clear that the capacity to define the real advancement of the microvascular involvement during SSc evolution, either in the diffuse or in the limited subset, will be significant for the choice of treatment (immunosuppressive, vasodilatory, vasoactive and its combination, future targeted therapies) to eventually achieve disease remission.

## Abbreviations

EC: Endothelial cell
SSc: Systemic sclerosis
VEGF: Vascular endothelial growth factor
